# Chylothorax due to central vein thrombosis treated by venous stenting using a dual approach: A case report

**DOI:** 10.1097/MD.0000000000028100

**Published:** 2021-12-10

**Authors:** Arthur Bouche, Jean-Francois De Wispelaere, Françoise Kayser, Elodie Collinge, Hadrien Fourneau

**Affiliations:** a Department of Radiology, CHU UCL Namur/Site Godinne, Yvoir, Belgium; b Department of Hematology, CHU UCL Namur/Site Godinne, Yvoir, Belgium.

**Keywords:** brachiocephalic thrombosis, chylothorax, COVID-19, stenting

## Abstract

**Rationale:**

Central vein thrombosis is an uncommon cause of chylothorax, usually secondary to central venous catheterization in association with prothrombotic state causes such as malignancies. In the following case, thrombosis was located in the left brachiocephalic vein and caused recurrent chylothorax resistant to the first line of treatment and successfully treated by percutaneous recanalization using a dual approach.

**Patient concerns:**

A 52-year-old male patient with current follicular lymphoma undergoing treatment and recent history of COVID-19 pulmonary infection was hospitalized for dyspnea. A chest X-ray revealed extensive bilateral pleural effusion. Analysis of the pleural fluid was compatible with a chylothorax. Iodin injected thoracic computed tomography (CT) revealed a complete left brachiocephalic thrombosis extending to the left axillary vein, with no thoracic mass.

**Diagnoses:**

Chylothorax due to left brachiocephalic vein thrombosis.

**Interventions:**

Following an unsuccessful first line of treatment consisting of a low-fat diet, somatostatins and anticoagulation medication, the patient was elected to undergo minimally invasive venous recanalization with stenting. After a first failed attempt of recanalization by femoral access, we successfully crossed the thrombus through brachial access and conducted a dilatation and stenting of the brachiocephalic vein by femoral access, using a “telepheric” method.

**Outcomes:**

During the 4-month follow up, PET-scanner and chest X-ray demonstrated a significant reduction of the pleural effusion, and the patient reported complete clinical recovery.

**Lessons:**

Central vein thrombosis is an unusual cause of chylothorax. We report a case of chylothorax complicating a brachiocephalic vein thrombosis successfully treated by percutaneous recanalization and stenting using a dual brachial and femoral approach. No thoracic duct embolization or ligature was required.

## 1 Introduction

Chylothorax is a type of pleural effusion defined by a pleural fluid triglyceride level greater than 110 mg/dL.^[1,2]^ It is an uncommon condition due to the leakage of chyle from the lymphatic system to the pleural cavity resulting in most cases from direct injury or obstruction of the thoracic duct (TD).^[2–4]^ Patients may present with shortness of breath as well as malnutrition in chronic forms.^[2]^ Treatment modality is decided based on the severity of symptoms and conservative treatment consists primarily in a low-fat diet to which intravenous infusion of somatostatin may be added.^[5]^ Secondarily, the cause of chylothorax must be treated in case of obstructive etiologies such as the presence of malignant mediastinal tumors.^[2]^ Central vein thrombosis is a rare cause of chylothorax, especially in the adult population.^[2,4,6]^ We report a case of left brachiocephalic thrombosis causing symptomatic bilateral chylothorax treated successfully by recanalization and stenting.

## 2 Case presentation

**Figure 1:**
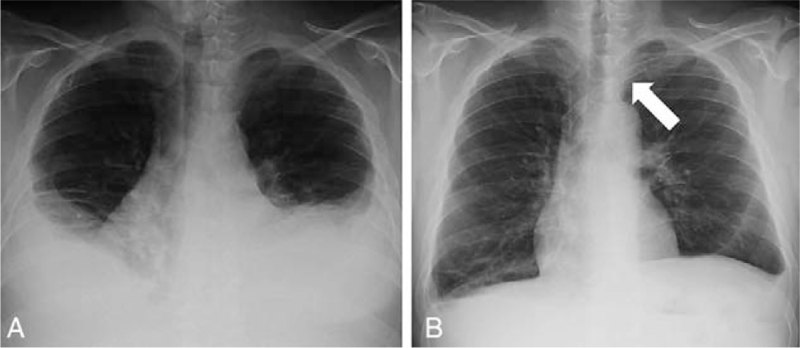
(A) Chest X-ray performed at the admission showing a bilateral pleural effusion. (B) Chest X-ray performed 1 month after the intervention showing near resolution of pleural effusions. The stent in the left brachiocephalic vein is clearly visible (arrow).

**Figure 2:**
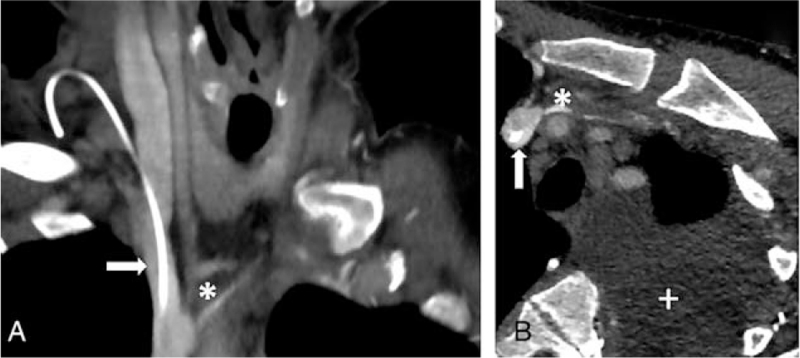
Injected CT scan performed at admission (A: coronal reconstruction, B: corrected axial reconstruction). The thrombus within the left brachiocephalic vein is well individualized (∗), ending at the junction between this vein and the superior vena cava (arrow). Presence of a large pleural effusion, a chylothorax in our case (+).

A 52-year-old male patient, with a 2-month history of follicular lymphoma treated by chemotherapy, was admitted in our institution for dyspnea. He had also presented a month earlier with a COVID-19 pulmonary infection. A chest X-ray revealed a significant bilateral pleural effusion (Fig. 1A). Thoracocenthesis and subsequent analysis of the pleural fluid confirmed the chylous nature of the effusion. Consequently, a thoraco-abdominal iodin injected Computed Tomography (CT) in portal phase was performed to determine the underlying cause. It demonstrated a left brachiocephalic veinous thrombosis extending to the distal portion of the axillary vein with no adenopathy along the thoracic duct pathway (Fig. 2A and 2).

After multidisciplinary discussion, the patient was referred to our interventional radiology department for brachiocephalic recanalization and stenting following the unsuccessful conservative treatment by low-fat diet and somatostatins combined with low molecular weight heparin (LMWH).

**Figure 3:**
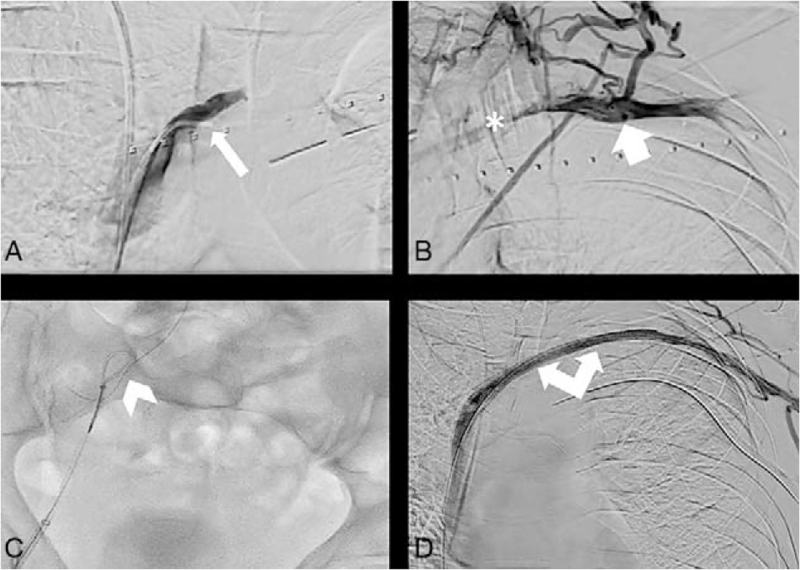
Scopic images from the interventional radiology procedure. (A) Phlebography with a 4F cobra catheter (arrow) located at the ostium of the left brachiocephalic vein showing an absence of opacification of the lateral 2/3 of the vein. (B) Phlebography with a 4F cobra catheter (large arrow) located in the lateral 1/3 of the left brachiocephalic vein showing the thrombus located in the internal part of this latter one (∗). (C) Capture of the long guidewire by a snare kit in order to tract it through the femoral sheath (arrowhead). (D) Final phlebography through the brachial sheath showing a re-permeabilization of the stented brachiocephalic vein (double arrow).

Under local anesthesia, ultrasound guided femoral access was obtained with a 7F sheath. Superior cava vein and ostium of the left brachiocephalic were catheterized with a 0.035 inch guide combined with a Cobra 4F catheter. Venography confirmed the left brachiocephalic thrombosis (Fig. 3A). Following an unsuccessful repeated attempt to cross the thrombosis, the decision was made to undertake a left brachial venous access using a 4F sheath (Fig. 3B). This new approach granted an easy crossing of the thrombosis using the same guide and catheter. A 4-m-long 0.035 inch guide was then pushed from the brachial venous access to the femoral vein and a “bridge” was established between the two venous accesses using an Atrieve Vascular Snare Kit to capture the guide and tract it through the femoral venous sheath (Fig. 3C). Venous dilatation was carried out on the long guide using an 8 mm Mustang balloon by femoral access and stenting was achieved with a veinous Wall stent 10 × 94 mm. Post-stenting venography was performed and demonstrated brachiocephalic vein re-permeabilization (Fig. 3D). The patient had no post-operative complications and the symptomatology quickly resolved with a significant reduction of the pleural effusion on following PET-scanner and Chest X-ray control (Fig. 1B).

## 3 Discussion

Anatomical understanding of the lymphatic system is crucial for identifying plausible etiologies of obstructive or disruptive chylothorax. Carrying 1 to 2 L of fluid a day, the TD is the largest lymphatic duct in the body. In most cases, it emerges from the aortic hiatus and is located between the azygos vein and the descending aorta. It then drains into the left veins of the neck. Subsequently, obstruction of the brachiocephalic left vein will cause increased backpressure in the TD, resulting in chylothorax through the same mechanism as direct TD obstruction.^[7]^ In the present case, the absence of any other identifiable etiology, in particular a mediastinal lymphoma mass, leads to expect a complete resolution of the pleural effusions following brachiocephalic left vein recanalization.

To the best of our knowledge, very few cases of chylothorax caused by isolated brachiocephalic thrombosis and treated by angiographic recanalization were reported. Most of the time, interventional radiological treatment of chylothorax involves a procedure on the lymphatics, such as TD embolization.^[8]^ In our case, chylothorax was successfully treated with exclusive brachiocephalic recanalization and stenting in a relatively young patient avoiding complications and secondary effects of TD embolization or surgical ligature such as chronic diarrhea and lower extremities swelling.^[9]^

Failure to cross the thrombus using a single femoral approach was due to the extent and position of the thrombus, ending at the ostium between the brachiocephalic vein and the superior cava vein. Using a brachial approach allowed us an easy crossing of the brachiocephalic thrombus. However, the stenting device could not be introduced from the brachial access since the smaller caliber of the vein could lead to injury. Therefore, we used a dual or “telepheric” approach: a long 0.035 inch guide was pushed from the brachial access to the femoral vein and was captured by a lasso system in order to tract it through the femoral 7F access. The balloon dilatation was performed from the femoral 7F sheath along this guide, allowing for the safe introduction and removal of the stenting device.

## 4 Conclusion

Central vein thrombosis is an unusual cause of chylothorax. We report a case of chylothorax complicating a brachiocephalic vein thrombosis successfully treated by percutaneous recanalization and stenting using a dual brachial and femoral approach. No thoracic duct embolization or ligature was required.

## Author contributions

AB was the main author of the manuscript. HF was the major contributor in writing the manuscript and one of the performer of the procedure. JDW revised it and was one of the performer of the procedure. FK and EC contributed to the writing. All authors read and approved the final manuscript.

**Supervision:** Jean-François De Wispelaere, Hadrien Fourneau.

**Writing – original draft:** Arthur Bouche, Elodie Collinge, Hadrien Fourneau.

**Writing – review & editing:** Françoise Kayser, Hadrien Fourneau.
